# *FUS* P525L mutation causing amyotrophic lateral sclerosis and movement disorders

**DOI:** 10.1002/brb3.1625

**Published:** 2020-04-19

**Authors:** Binbin Zhou, Huan Wang, Yu Cai, Han Wen, Lulu Wang, Min Zhu, Yunqing Chen, Yanyan Yu, Xi Lu, Meihong Zhou, Pu Fang, Xiaobing Li, Daojun Hong

**Affiliations:** ^1^ Department of Neurology The First Affiliated Hospital of Nanchang University Nanchang China; ^2^ Department of Diagnostic Center Ascension Seton Medical Center Austin Austin TX USA

**Keywords:** amyotrophic lateral sclerosis, fused in sarcoma, lipid droplet, movement disorder, tremor

## Abstract

**Background:**

Mutations in the fused in sarcoma (FUS) gene have been associated with amyotrophic lateral sclerosis (ALS), frontotemporal lobar degeneration, and essential tremor. Among the *FUS* mutations, p.P525L as a hot spot variant has been reported in more than 20 patients with ALS. Apart from the typical ALS phenotype, patients with p.P525L mutation exhibit some atypical symptoms. However, movement disorders related to p.P525L mutation have not been emphasized currently.

**Methods:**

Two unrelated patients with ALS were evaluated through a set of clinical and laboratory tests. The genetic screening was performed through next‐generation sequencing. Muscle biopsies were performed on the 2 patients. Muscle samples were stained according to standard histological and immunohistochemical procedures.

**Results:**

The first patient presented with juvenile‐onset neurogenic weakness and wasting and simultaneously had dropped head, ophthalmoplegia, tremor, involuntary movements, and cognitive impairments. The second patient showed a typical ALS phenotype and prominent adventitious movements. Genetic screening disclosed de novo p.P525L *FUS* mutation in the 2 patients by family cosegregation analysis. Muscle biopsy showed neurogenic patterns and numerous lipid droplets aggregating in the fibers.

**Conclusion:**

Apart from the typical ALS phenotype, patients with p.P525L mutation in the *FUS* gene can present with great clinical heterogeneity including multiple movement disorders. Numerous lipid droplets in muscle fibers indicate that skeletal muscle is likely an important therapeutic target for ALS.

## Graphical abstract

Besides the typical ALS phenotype, patients with p.P525L mutation in the FUS gene can present with great clinical heterogeneity including multiple movement disorders. Numerous lipid droplets in muscle fibers indicate that skeletal muscle is likely an important therapeutic target for ALS.

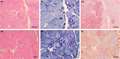

## 1 INTRODUCTION

Amyotrophic lateral sclerosis (ALS) is a fatal neurodegenerative disease pathologically characterized by aggressive deterioration of frontotemporal neurons, corticospinal tract, brainstem neurons, and anterior horn neurons (Statland, Barohn, McVey, Katz, & Dimachkie, 2015). Typical symptoms of ALS present with weakness and wasting of the limbs, bulbar paralysis, and respiratory insufficiency. Approximately 90% of patients are sporadic ALS (SALS), and the remaining 10% of patients are familial ALS (FALS) (Chia, Chiò, & Traynor, 2018). Most patients with ALS have adult onset of age with a peak age at onset of 47–52 years for FALS and 58–63 years for SALS (Kiernan et al., 2011).

To date, there are nearly 30 genes that are responsible for genetic causes of ALS or are highly correlated with ALS. Since two academic groups simultaneously identify the fused in sarcoma (FUS) gene as one of genetic causes of ALS (Kwiatkowski et al., 2009; Vance et al., 2009), more than 100 *FUS* mutations have been recorded in the Human Gene Mutation Database (Shang & Huang, 2016). FUS is an RNA‐binding protein whose genetic mutations or pathological inclusions are currently associated with multiple neurological diseases including ALS (Vance et al., 2009), frontotemporal lobar degeneration (FTLD; Cairns & Ghoshal, 2010), and essential tremor (ET; Merner et al., 2012), although the results of ET in most confirmatory studies are negative (Zheng et al., 2013). A meta‐analysis including 154 ALS cases with *FUS* mutations shows an average disease onset of 43.8 ± 17.4 years. More than 60% of cases with *FUS* mutations show disease onset younger than 45 years of age, evenly many juvenile‐onset ALS cases present with disease course in their late teens or early 20s (Huang et al., 2010).

Most patients with *FUS* mutations show a typical ALS phenotype, but some patients display a variety of accompanying symptoms including parkinsonism‐like symptoms (Yan et al., 2010), myoclonic jerks (Dodd, Power, Ealing, & Hamdalla, 2019), and peripheral neuropathy (Mackenzie et al., 2011). Among the *FUS* mutations, p.P525L mutation as a hot spot variant has been reported in more than 20 patients with ALS. Besides the typical symptoms, the phenotype associated with p.P525L mutation concomitantly exhibits some atypical symptoms such as developmental delay (Mochizuki et al., 2012), ophthalmoplegia (Leblond et al., 2016), cognitive impairments (Yan et al., 2010), and tremor (Eura et al., 2019). In this study, we describe two patients with *FUS* p.P525L mutation presenting with rapid progressive ALS and multiple movement disorders, which indicate the great clinical heterogeneity associated with *FUS* p.P525L mutation.

## 2 MATERIALS AND METHODS

### 2.1 Subjects

Patients were recruited at the neurological department of the first affiliated hospital of Nanchang University from January 2018 to December 2019. ALS was diagnosed on a clinical and electrophysiological basis of the revised El Escorial criteria. Initial symptoms, age of onset, progression of disability, and clinical manifestations were recorded as reported by the patients and their relatives. Time of disease duration was calculated from the reported date of symptom onset to the initial medical consultation in our department. All samples and medical data were obtained after a written consent signed by each individual in compliance with the bioethical laws of China as well as the Declaration of Helsinki. The research was also approved by the ethics committee of the first affiliated hospital of Nanchang University.

### 2.2 Genetic testing

Genomic DNA was extracted from peripheral blood samples. Targeted exon enrichment was performed using SureSelect Human All Exon V5 (Agilent Technologies). The exon‐enriched DNA libraries were subjected to paired‐end sequencing with the Hiseq2000 platform (Illumina, Inc.). Sequence data were mapped with BWA and SAMTOOLS onto the hg38 human genome as a reference. Calls with variant quality less than 20 were filtered out, and 95% of the targeted bases were covered sufficiently to pass our thresholds for calling single nucleotide polymorphisms (SNP), nonsynonymous/splice acceptor and donor site, insertions or deletions (NS/SS/InDel) variants in the dbSNP v137, ESP6500, and 1,000 Genome were removed. Synonymous changes were filtered using SIFT software (http://sift.jcvi.org). Sanger sequencing with specific primers was conducted to confirm the *FUS* mutation in the patients and their family members (parents and siblings). Analysis of the hexanucleotide repeats in *C9orf72* and the trinucleotide repeats in the Huntingtin (*HTT)* gene was performed by a repeat‐primed polymerase chain reaction (RP‐PCR).

### 2.3 Muscle pathological examination

Muscle biopsies were performed from the left bicep of the two cases. The muscle tissue was frozen and then cut at 8‐μm sections. These sections were stained according to standard histological and enzyme histochemical procedures with hematoxylin and eosin (H&E), modified Gomori trichrome (MGT), periodic acidic Schiff (PAS), oil red O (ORO), nicotinamide adenine dinucleotide tetrazolium reductase (NADH‐TR), succinate dehydrogenase (SDH), cytochrome *c* oxidase (COX), nonspecific esterase (NSE), and ATPase stain. Monoclonal antibody of FUS (Abcam) was used to detect the distribution of FUS protein in the muscle specimens by immunohistochemical stain.

## 3 RESULT

### 3.1 Clinical features

#### 3.1.1 Case 1

**FIGURE 1:**
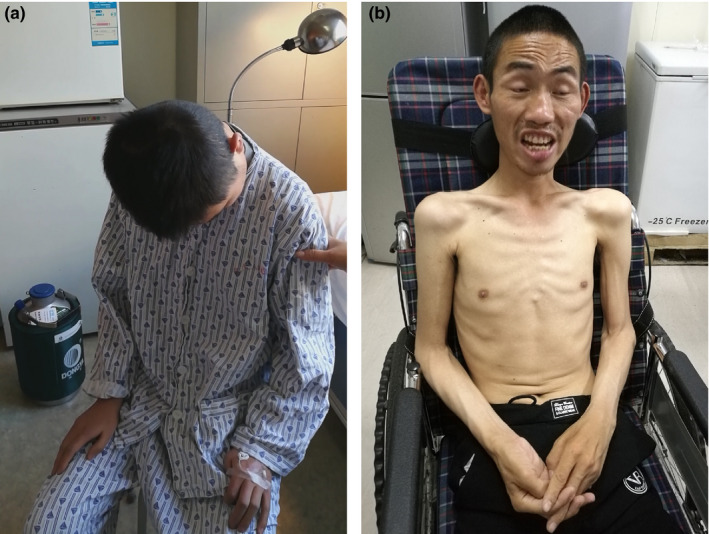
The clinical pictures of the two patients. Patient 1 is a 19‐year‐old young man and presents with a dropped head (a). Patient 2 is a 34‐year‐old man and shows muscle wasting and adventitious movements (b)

A 19‐year‐old young man was referred to our department with a history of cervicodorsal pain and dropped head for 3 months (Figure 1a), chin and both hands tremor for 2 months, and muscle weakness of bilateral upper limbs for 1 month. He had a learning disability from childhood so that he could not accomplish the curriculum of junior middle school. He also showed difficulty in getting along with his classmates and workmates. Physical examinations on admission revealed that his eyeballs presented with esotropia and mild limitation of abduction. He showed a dropped head with muscle strength of neck extension grading 1/5 according to the Medical Research Council scale. Obvious tremors were observed in the chin and both hands, which worsen when he made intentional activity or got nervous (Video S1). Involuntary dorsiflexion of right foot was occasionally observed. He showed mild facial muscle weakness and mild atrophied tongue with fasciculation, but no signs of bulbar paralysis. Muscle strength was 3 grade in the proximal upper limbs, 4 grade in the distal upper limb, 5 grade in the proximal lower limbs, and 5 grade in the distal lower limbs. Deep tendon reflexes were increased, but pathological reflexes were negative. There was no evidence of parkinsonism, sensory disturbance, ataxia, or autonomic dysfunction. The blood count, blood biochemistry, thyroid function, parathyroid hormone, metal toxin analysis, blood acylcarnitines and urine organic acid profiles, ganglioside antibody spectrum, paraneoplastic antibody spectrum, and antibodies of autoimmune encephalitis were all negative to abnormalities. Cerebrospinal fluid examinations revealed a mild increase of protein level 0.64 g/L (normal 0.15–0.45 g/L) with normal other indexes. Brain and spinal MRI were normal. Lung function revealed severely decreased forced vital capacity (FVC; 34.9%) and vital capacity (VC; 33.5%). The results of a nerve conduction study were normal, but needle electromyography showed acute and chronic denervation in four segments.

#### 3.1.2 Case 2

A 34‐year‐old man was referred to our department with progressive limb weakness for 6 months, adventitious movements for 5 months, and dysphagia for 2 months (Figure 1b). The man had no family history of other neurological diseases including muscle weakness and extrapyramidal disorders. Physical examination on admission revealed that an emaciated man presented with adventitious movements characterized by eyebrows squeezing, neck torsion, torso, and limb twisting. The movements involved all body parts including the face, tongue, neck, torso, and limbs. These movements were repetitive, but not rhythmic, stereotyped, or high speed, flowing from side to side, from limb to limb, and merging into the appearance of continuous motion (Video S2). The movements tended to worsen with stress or excitement and relieve during sleep. He had mild facial muscle weakness, dysarthria, dysphagia, and atrophied tongue with fasciculation. The muscle strength was 3 grade in the proximal upper limbs, 1 grade in the left hand and 3 grade in the right hand, 3 grade in the proximal lower limbs, and 2 grade in the distal lower limbs. Deep tendon reflexes of lower limbs were increased, but pathological reflexes were absent. There was no evidence of sensory disturbance or autonomic dysfunction. The blood count, morphology of erythrocyte, blood biochemistry, ceruloplasmin, thyroid function, parathyroid hormone, metal toxin analysis, ganglioside antibody spectrum, paraneoplastic antibody spectrum, and antibodies of autoimmune encephalitis were normal. Cerebrospinal fluid examinations were negative. The cerebral CT was normal. Lung function revealed a decreased forced vital capacity (FVC; 65.3%). The results of a nerve conduction study revealed a mild decrease of compound muscle action potentials (CMAP) in the left median nerve (4.6 mv) and left peroneal nerve (1.9 mv). The needle electromyography showed acute or chronic denervation in four segments.

### 3.2 Myopathological changes

**FIGURE 2:**
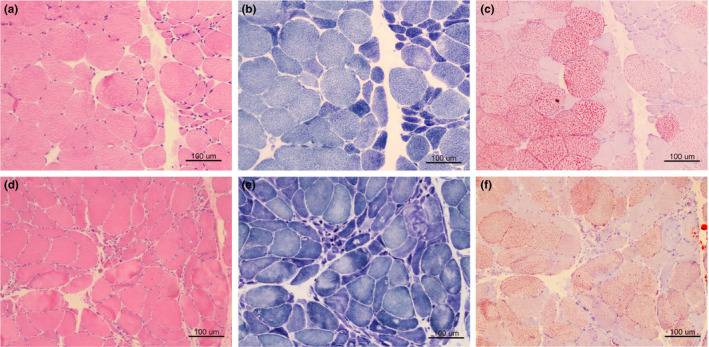
The myopathological features of the two patients. Muscle biopsy shows grouping of small angular atrophic fibers on HE stain, suggesting a neurogenic process (a: case 1; d: case 2). NADH stain reveals dark angular fibers and target fibers (b: case 1; e: case 2). Numerous lipid droplets aggregate in the relatively hypertrophy fibers on ORO stain (c: case 1; f: case 2)

Muscle specimens of both patients showed grouping of small angular atrophic fibers, increased variation of fiber size, and mild proliferation of connective tissue, suggesting a neurogenic process (Figure 2a,d). Some fibers exhibited target fibers on NADH stain (Figure 2b,e). The ATPase stain confirmed the angular fibers involving in two fiber types. Numerous lipid droplets were aggregated in the relatively hypertrophy fibers on ORO stain (Figure 2c,f), especially in the specimen of case 1. The immunohistochemical stain showed that the FUS‐positive materials only deposited in the nucleus, and no inclusions were found in the cytoplasm of muscle fiber. On the basis of these findings, we administered case 1 for riboflavin (50 mg tid) and levocarnitine (1 g, bid). The patient can partially lift his head one week later (Video S3), but no further benefits were observed one month later.

### 3.3 Genetic mutation

**FIGURE 3:**
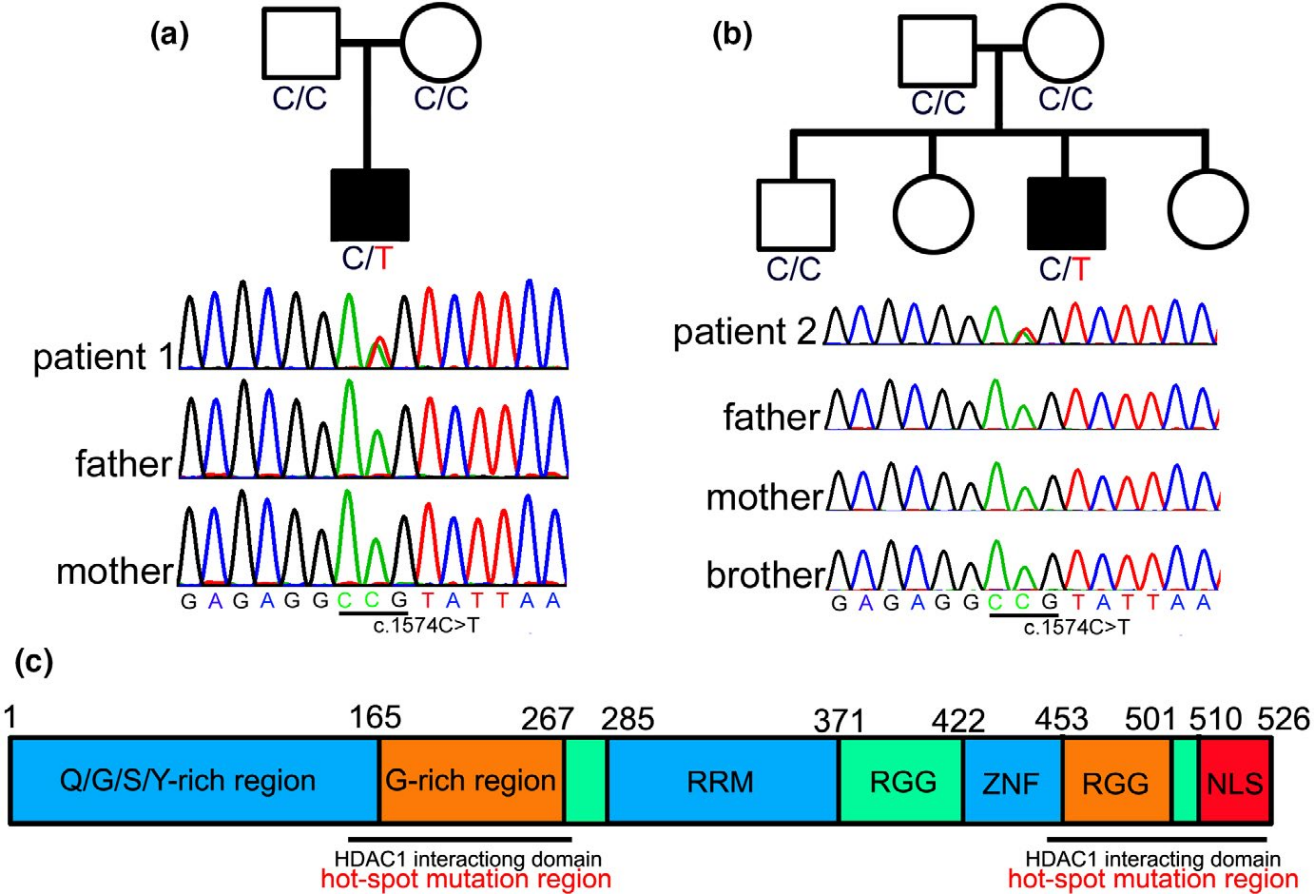
Genetic mutation of the patients. Genetic sequencing of patient 1 (a) and patient 2 (b) disclosed a mutation with c.1574C>T (p.P525L) in the *FUS* gene, while their parents or brother do not carry the variant, indicating the variant is a de novo mutation. Full‐length human FUS protein can be divided into Q/G/S/Y domain, G‐rich region, RNA recognition motif (RRM), two Arg‐Gly‐Gly (RGG)‐repeat regions interrupted by a zinc finger motif (ZNF), and nuclear localization signal (NLS). Structure–function analyses have shown that the G‐rich domain (amino acids 156–262) and C‐terminal domain (amino acids 450–526) of FUS are required for interaction of FUS and histone deacetylase 1 (HDAC1), which harbor most of the ALS mutations (c)

As for case 1, exome sequencing disclosed a missense mutation with c.1574C>T (p.P525L) in the exon 15 of *FUS*. Sanger sequencing confirmed the heterozygous mutation in the patient, while the parent did not carry the variant, which indicated the variation was a de novo mutation (Figure 3a). The number of G_4_C_2_ repeats in *C9orf72* was 10 copies. No other causative mutations associated with ALS were identified in the case 1, while the exome sequencing revealed some variants of benign or uncertain significance including c.1340C>A (p.S447*) in the *ABCA7* gene associated with Alzheimer's disease, c.344C>T (p.T115M) in the *IGHMBP2* gene associated with distal spinal muscle atrophy, and c.231_243delGCAGCAGCAGCAG (p.Q77Hfs*63) in the *TBP* gene associated with Parkinson's disease (Table S1).

As for case 2, the number of CAG repeats of *HTT* was initially identified with 16 and 19 copies at the two allelic chromosomes, respectively. A whole‐genome sequencing was then performed and revealed a missense mutation with c.1574C>T (p.P525L) in the exon 15 of *FUS*. Sanger sequencing confirmed the heterozygous mutation in the patient, while the parent and brother did not carry the variant, indicating the variation was a de novo mutation (Figure 3b). A homology search in different species demonstrates that the proline at residue 525 is highly evolutionarily conserved (Figure 3c). The number of G_4_C_2_ repeats in *C9orf72* was 8 copies. No other causative mutations associated with ALS were identified in the case 1, while the exome sequencing revealed some variants of benign or uncertain significance including c.1321T>C (p.Y441H) in the *MAPT* gene associated with frontotemporal dementia, c.475G>A (p.E159K) in the *ALS2* gene associated with juvenile‐onset ALS, c.1844C>T (p.P615L) in the *NEFH* gene associated with Charcot–Marie–Tooth disease, and c.4445C>T (p.T1482I) in the *TRPM7* gene associated with ALS/parkinsonism/dementia susceptibility complex 1 (Table S2).

## 4 DISCUSSION

The clinical classification of ALS associated with *FUS* mutation has been designated as familial ALS type 6 (ALS6; Vance et al., 2009). Most patients with ALS6 appear to be predominantly lower motor neuron with young onset, aggressive course, high incidence of bulbar symptoms, and early respiratory involvement, although typical ALS phenotype and slower disease course have also been described in some patients (Naumann et al., 2020; Sproviero et al., 2012). *FUS* mutations have been reported to be responsible for 3%–4% of familial ALS and for less than 1% of sporadic ALS (Zou et al., 2013). Among these mutations, p.P525L *FUS* mutation has been reported to be consistently associated with younger onset, rapid disease course, and high proportion of de novo mutations in sporadic patients (Conte et al., 2012). We summarized the demographic data and clinical features of all reported patients with p.P525L/R mutation (Table 1; Bäumer et al., 2010; Chiò et al., 2009; Conte et al., 2012; Deng et al., 2019; Eura et al., 2019; Fecto & Siddique, 2011; Huang et al., 2010; Hübers et al., 2015; Ito et al., 2011; King et al., 2015; Kuang et al., 2017; Kwiatkowski et al., 2009; Leblond et al., 2016; Mochizuki et al., 2012; Naumann et al., 2020; Sproviero et al., 2012; Zou et al., 2013). The mean age of onset in all patients with p.P525L/R mutation was 21.0 ± 8.1 years, and all patients progressed very rapidly, with an average interval of 13.8 months from onset to death or tracheotomy. Besides the typical ALS phenotype, a wide variety of accompanying symptoms had been reported in those patients with p.P525L mutation. Interestingly, our patients had several novel findings as compared with reported FUS‐ALS patients with p.P525L mutation.

**TABLE 1:** The clinical summarization of ALS patients with mutation at 525 proline residue

Literature	Case	Sex	Age onset (year)	Survival (m)	De novo	Onset site	Bulbar	Limb	UMN	Other symptoms	Mutation
Kwiatkowski et al. (2009)	1	UA	22	6	UA	UA	UA	UA	UA	UA	p.P525L
Kwiatkowski et al. (2009)	2	UA	44	UA	UA	UA	UA	UA	UA	UA	p.P525L
Kwiatkowski et al. (2009)	3	UA	15	UA	UA	UA	UA	UA	UA	UA	p.P525L
Chiò et al. (2009)	4	F	21	12	No	Bulbar	Yes	Yes	Yes	No	p.P525L
Bäumer et al. (2010)	5	F	22	10	Yes	LE	No	Yes	No	No	p.P525L
Bäumer et al. (2010)	6	F	18	11	Yes	UE	Yes	Yes	Yes	No	p.P525L
Huang et al. (2010)	7	F	13	17	Yes	LE	No	Yes	No	Developmental delay, learning difficulty	p.P525L
Ito et al. (2011)	8	M	13	24	No	LE	UA	Yes	UA	No	p.P525L
Fecto and Siddique (2011)	9	UA	11	UA	No	UA	UA	UA	UA	No	p.P525L
Mochizuki et al. (2012)	10	F	13	15	No	limb	Yes	Yes	UA	Developmental delay	p.P525L
Sproviero et al. (2012)	11	M	26	13	Yes	Limb	Yes	Yes	No	No	p.P525L
Sproviero et al. (2012)	12	F	45	42	Yes	Limb	Yes	Yes	Yes	Multiple sclerosis	p.P525L
Zou et al. (2013)	14	F	19	UA	Yes	Limb	Yes	Yes	Yes	No	p.P525L
Conte et al. (2012)	13	F	11	14	Yes	Limb	No	Yes	Yes	No	p.P525L
Hübers et al. (2015)	15	F	18	UA	Yes	Bulbar	Yes	UA	UA	No	p.P525L
Hübers et al. (2015)	16	M	20	UA	Yes	Bulbar	Yes	UA	UA	No	p.P525L
Hübers et al. (2015)	17	M	24	7	Yes	Bulbar	Yes	UA	UA	No	p.P525L
King et al. (2015)	18	F	23	8	No	UA	No	Yes	Yes	No	p.P525L
Leblond et al. (2016)	19	F	21	6	Yes	Bulbar	Yes	Yes	Yes	Ophthalmoplegia	p.P525L
Kuang et al. (2017)	20	F	26	12	No	UE	Yes	Yes	Yes	No	p.P525R
Eura et al. (2019)	21	F	19	6	Yes	Bulbar	Yes	Yes	Yes	Autism, tremor	p.P525L
Deng et al. (2019)	22	F	16	UA	No	Spinal	Yes	UA	UA	UA	p.P525L
Deng et al. (2019)	23	M	19	UA	No	Bulbar	Yes	UA	UA	UA	p.P525L
Deng et al. (2019)	24	M	22	UA	No	Spinal	Yes	UA	UA	UA	p.P525L
Naumann et al. (2020)	25	F	22	UA	UA	Arms	UA	Yes	UA	No	p.P525L
Naumann et al. (2020)	26	F	17	24	UA	Bulbar	Yes	UA	UA	No	p.P525L
Naumann et al. (2020)	27	M	17	15	UA	Legs	No	Yes	UA	No	p.P525L
This study	28	M	19	7	Yes	Limb	No	Yes	Yes	Ophthalmoplegia, tremor, developmental delay, learning difficulty, adventitious movements	p.P525L
This study	29	M	34	13	Yes	Bulbar	Yes	Yes	Yes	adventitious movements	p.P525L

Abbreviations: F: female; LE: lower limb; M: male; UA: unavailable; UE: upper limb; UMN: upper motor neuron.

The first patient presented with chin and hands tremor, which aggravated when he made intentional activity or an effort to maintain posture. *FUS* p.Gln290* mutation had been reported to be exclusively responsible for ET in a large Italian family without ALS symptoms, and the pathogenesis was supposed to be associated with a *FUS* nonsense‐mediated decay pathway (Merner et al., 2012). However, multiple validating studies subsequently revealed rare exonic variants in *FUS* were not more frequent in ET than in the general population, indicating no evidence for a role of rare genetic variants in the pathogenesis of ET (Zheng et al., 2013), apart from the initially published *FUS* mutation segregating in the large ET family. Recently, a 19‐year‐old Japanese FUS‐ALS patient with p.P525L mutation displayed a rapid‐onset ALS co‐occurring with autism spectrum disorder and tremor (Eura et al., 2019). Similarly, our case showed a phenotype as those of Japanese girl with juvenile‐onset ALS, tremor, and cognitive impairments possibly originating from frontotemporal dysfunction. In addition, our patient presented with ophthalmoplegia and involuntary dorsiflexion of right foot, indicating a widespread neurological dysfunction sparing of abnormalities in brain MRI. *FUS* mutations or pathological inclusions have been associated with neurological diseases including ALS, FTLD, and ET. The recurrent case indicated that the three entities associated with *FUS* mutation could simultaneously exhibit in a single patient, suggesting that the tremor associated with p.P525L mutation might be not a coincidental symptom, which also indicated that other pathogenic mechanisms than nonsense‐mediated decay pathway were responsible for the dominant dysfunction mutation.

The second patient was initially misdiagnosed as Huntington's disease due to the notable movements. A reviewer suggested that the adventitious movements were a sign of compensatory muscle activity in an effort to maintain posture or reposition a limb; however, we disagreed because the patient showed uncontrolled movements characterized by eyebrows squeezing, neck torsion, torso, and limb twisting. These movements were not high speed and great amplitude, and unlike chorea‐like movements, whereas the movements were involuntary and referring to hyperactivity possibly associated with extrapyramidal symptoms. The co‐occurrence of FUS‐ALS and hyperactive movements might be a coincidence, but might also indicate an underlying relationship between FUS dysfunction and extrapyramidal pathway. Aberrant protein folding is severely problematic and manifests in numerous disorders, including ALS, Huntington's disease, Parkinson's disease, and Alzheimer's disease. Patients with each of these disorders are characterized by the accumulation of mislocalized protein deposits (Deng et al., 2018). In addition, patients associated with protein accumulation caused by C9ORF72 hexanucleotide repeat expansion have exhibited either Huntington's disease or ALS, or both. Under this pathophysiological mechanism, the concurrence of ALS phenotype and motion hyperactivity phenotype may be reasonable in patients with *FUS* p.P525L defects that trigger the dysfunction of nuclear mass transport and protein deposits (Koutsis, Karadima, Kartanou, Kladi, & Panas, 2015).

FUS is one of the major components of nuclear polyQ aggregate‐interacting proteins in Huntington's disease and was also associated with neuronal intranuclear inclusions (Kino et al., 2016). Because FUS has a variety of functional roles, the aggregating FUS may play a role in diverse pathological changes in the brains of patients with ALS6 and induce neurodegeneration of multiple systems including the frontal lobe, the basal ganglia, substantia nigra, cerebellum, and other related areas (Mochizuki et al., 2012). Compared with previously reported ALS6 cases, the prominent movement disorders were the characteristic features in both patients, indicating that *FUS* mutation can affect the function of movement coordinating system in addition to motor neuron degeneration. It is open to discuss if this is a coincidental finding or linked to the identified mutation. Given the oligogenic nature of ALS, we also identified several variants associated with ALS‐FTD, Parkinson's disease, and Alzheimer's disease through exome sequencing. These variants might act as disease modifiers, whereas the variants were benign or uncertain significance, and the oligogenic relationship would be difficult to be clearly evaluated.

One very interesting clinical feature observed in patient with *FUS* mutation was a severely disabling dropped‐head syndrome, which emerged as the initial symptom in our first patient. Primary dropped‐head syndrome is usually caused by cervical extensor myopathy of unknown etiology, while secondary dropped‐head syndrome is mostly associated with motor neuron disease (Peng et al., 2015). Our patient with p.P525L mutation showed a neck pain and a significant decrease of cervical extensor muscle strength, which have not been reported in previous cases. Patients with mutations at other FUS loci (e.g., at position 521) also showed axial muscle atrophy and profound weakness as previously reported (Blair et al., 2010; Naumann et al., 2020). So this phenotype is likely to be characteristic of individuals with C‐terminal FUS mutations.

The muscle biopsy specimens of the two patients showed acute or subacute neurogenic processes with angular fibers grouping and target‐like fibers, which were consistent with the results of previous studies (King et al., 2015). Eura *et al.* had observed the muscle pathology in an ALS patient with p.P525L *FUS* mutation and found no abnormal inclusions or aggregations (Eura et al., 2019). We neither observed aggregation of FUS inclusions in the two patients with p.P525L *FUS* mutation, suggesting p.P525L mutation directly unaffected muscle pathology. ALS patients' or models’ muscles exhibited increased reliance on fatty acids in physiological studies, while the extent of lipid storage in ALS muscles was rarely elucidated (Palamiuc et al., 2015). To our surprise, we found that numerous lipid droplets were deposited in the relatively hypertrophy fibers. Therefore, the patient 1 was given riboflavin and levocarnitine to modify the lipid metabolism and got a partial improvement. Animal model with p.P525L mutation exhibited that mutant FUS disrupted assembly and function of the mitochondrial ATP synthase complex and possibly resulted in irreparable mitochondrial damage that might cause impediment of lipid metabolism pathway (Deng et al., 2018). We provided evidence that muscle metabolic alterations occurred in motor neuron degeneration, indicating that skeletal muscle was likely an important therapeutic target for ALS.

Full‐length human FUS protein can be divided into Q/G/S/Y domain, G‐rich region, RNA recognition motif (RRM), two Arg‐Gly‐Gly (RGG)‐repeat regions interrupted by a zinc finger motif (ZNF), and nuclear localization signal (NLS) (Shang & Huang, 2016). Structure–function analyses have shown that the G‐rich domain (amino acids 156–262) and C‐terminal domain (amino acids 450–526) of FUS are required for interaction of FUS and histone deacetylase 1 (HDAC1) (Shang & Huang, 2016), which harbor most of the ALS mutations (Figure 3c). The high frequency of de novo p.P525L mutation may be associated with the high GC content in the NLS domain, also be associated with a reduced life expectancy or reduced reproductive fitness. Overall, p.P525L mutation leads to a stronger cytosolic mislocalization of FUS protein, consistent with the phenotype of an earlier age of onset and a more aggressive disease process in the patients with the mutation (Ito et al., 2011).

In conclusion, *FUS* mutations may affect a broader range of functions in addition to degeneration of motor neurons. Apart from the typical ALS phenotype, patients with p.P525L mutation in the *FUS* gene can present with great clinical heterogeneity including tremor, movement disorders, dropped‐head syndrome, and cognitive impairments. Numerous lipid droplets in muscle fibers indicate that skeletal muscle is a possible therapeutic target for ALS.

## CONFLICT OF INTEREST

The authors declare that they have no competing interests.

## AUTHOR CONTRIBUTION

ZB, WH, WH, and WL contributed to the acquisition and analysis of data. ZM, CY, and YY performed the genetic analysis. LX, ZM, and LX performed the pathological study. FP and CY contributed to critical revision of the manuscript. ZB and HD contributed to the study design and drafted the manuscript.

## Supporting information

Table S1

Table S2

Video S1

Video S2

Video S3

## Data Availability

All relevant data are within the paper and its Supporting Information files.
